# Uses of Spices Amongst Generation Z Students at a University Located in Rural Poland: An Exploratory Study

**DOI:** 10.3390/nu18132139

**Published:** 2026-07-02

**Authors:** Agnieszka Panasiuk, Kamil K. Hozyasz

**Affiliations:** 1 Department of Dietetics, Faculty of Health Sciences, John Paul II University in Biała Podlaska, Sidorska Street 95/97, 21-500 Biała Podlaska, Poland; 2 Department of Nursing, Faculty of Health Sciences, John Paul II University in Biała Podlaska, Sidorska Street 95/97, 21-500 Biała Podlaska, Poland; khozyasz@gmail.com

**Keywords:** spices, health properties, non-communicable chronic diseases, prevention, students, Generation Z, Polish cuisine, Poland

## Abstract

**Background:** According to well-known dietary recommendations, herbs and spices are part of a healthy, balanced diet, and their consumption may contribute to improved health. Globalisation fosters greater exposure to other cultures and cuisines, including the use of spices. This study aimed to assess the awareness and attitudes towards spices among Polish students in a rural area. **Methods:** A survey study was conducted among 278 Generation Z students (aged 18–28 years old) from a university located in a small town in southeastern Poland. Questions concerning, a.o., preparing meals, awareness of spices’ properties, and the use of seasoning were included. **Results:** Most of the respondents declared using a lot of spices beyond salt and pepper (61.2%), more often women than men (67.9% vs. 45.1%; *p* = 0.0004), and more often participants aged ≥23 years than ≤22 years (82.9% vs. 58.0%; *p* = 0.005). Participants who grew their own spices and often watched TV culinary programs used more seasonings (72.4%; *p* = 0.001 and 85.2%; *p* = 0.0002, respectively). Less than half of the respondents (45%) recognised health properties in some of the spices, and 28.1% of them recognised health properties in spices in general, with more older participants (45.7%; *p* = 0.0402). Respondents with the highest awareness of the health properties of spices used them more often to improve their health (42.3%; *p* < 0.00). **Conclusions:** Exposure to cuisines from other cultures and their spices and the willingness to try new flavours among respondents were low. This might be due to sociodemographic factors, including origin from small, rural, traditional communities, where attachment to familiar recipes might be observed. Moreover, awareness of the healing benefits of spice use was low. Therefore, education about the composition and use of local spices might be helpful in increasing their intake for the benefit of health.

## 1. Introduction

A healthy lifestyle, including a balanced diet, affects overall well-being and prevents diseases such as diet-related non-communicable chronic diseases. Many recommended dietary patterns, including the Mediterranean diet, emphasise the importance of particular food product groups and their appropriate intake amounts, as well as the inclusion of herbs and spices [1]. They contribute to taste and flavour, as well as bioactive compounds, including polyphenols, which can be classified into different groups, such as phenolic acids, stilbenes, lignans, and flavonoids, with flavonoids being the largest group [2]. Phenolic compounds, found in herbs and spices, provide important benefits to the human body through a.o. antioxidant, anti-inflammatory, and anticancer properties [1,2,3]. It is worth noting that oxidative stress and inflammation are the major causes of a variety of chronic diseases; therefore, polyphenols, those found in spices, might have a protective effect against them [2]. For instance, turmeric contains curcumin, a polyphenol with a wide range of health benefits, including the aforementioned effects [4]. Spices such as cinnamon, garlic, ginger, mint, onion, and turmeric have beneficial effects on cardiovascular disorders. Bay leaves, black cumin, cinnamon, garlic, and turmeric are recommended for diabetes [4,5]. Turmeric, saffron, onion, mustard, garlic, and bay leaves have protective roles against cancer [5]. The therapeutic effects in obesity might be due to saffron and turmeric [5] as well as chilli due to the content of capsaicin, which might affect appetite reduction [6]. Regarding antimicrobial properties, spices also extend the shelf life of foods [6].

Global localisation and cultural factors determine the use of herbs and spices. Seasonings are widely used in global cuisines [1,7], including Polish cuisine [7]. Polish native plants, known since Ancient Slavic times, are a.o. caraway, juniper, nettle, and horseradish. Various culinary sources indicate the use of many herbs and spices in Poland since the medieval period, such as black pepper, oregano, thyme, cinnamon, marjoram, garlic, and parsley. Over the following centuries, Polish cuisine was enriched with more spices, facilitated by the border and trade with the Ottoman Empire and the settlement of Armenian and Greek merchants [7,8]. The deepening interest in the culture of Persia, including its culinary heritage, with saffron and barberry at the forefront, initiated the translation of Saadi’s “The Gulistan/The Rose Garden” into Polish by Samuel Otwinowski around 1610, which became the first translation in Europe. However, in the second half of the 20th century, political changes influenced the availability of food products and impacted cuisine in Poland and its impoverishment while it belonged to the Soviet Eastern Bloc [7,9,10].

Globalisation has fostered greater exposure to other cultures and their cuisines, which might influence eating behaviours, including the use of herbs and spices [7,11]. This applies in particular to the youngest generation, such as Generation Z (people born between 1995 and 2012 [11]), who grew up in the period of technological development, and with the Internet, which has shaped their worldview and behaviour [11]. In addition, the impact of Western culture has influenced values and lifestyle choices. Through globalisation, Generation Z can access information from all over the world, communicate and travel more easily, and be exposed to new concepts and various cultures. These results, at least nominally, are in a more diverse, open-minded, and globally connected generation [11,12]. Therefore, the younger generation is more likely to try new dietary trends or follow alternative diets, such as veganism and vegetarianism, because their food choices often result from reflective concerns about the ethical and sustainable aspects of food production [13].

The present study assessed the awareness of and attitudes towards spices, as well as their usage, among Polish Generation Z students in a rural settlement. It investigates the eating behaviours of the young population affected by globalisation and digital progress among a specific sociodemographic group, for which place of residence, attachment to tradition, and professed values may influence dietary choices.

## 2. Materials and Methods

The survey was conducted among 341 students from two faculties (Economic Sciences and Health Sciences) at John Paul II University in Biala Podlaska, Poland. It is a small town (approximately 55,000 inhabitants) located in the rural region of southeastern Poland, in the Lublin Voivodeship. The study protocol was approved by the Bioethics Committee of John Paul II University (Biała Podlaska, Poland).

The adapted questionnaire developed by Łuczaj et al. [7] was used in this study. It contains, a.o., questions concerning preparing meals, awareness of spices’ properties, and the use of seasoning. The inclusion criteria were place of origin (Polish students) and age (born between 1995 and 2012).

The study was conducted in October, during the winter term 2023/2024, via the paper-and-pencil mode after obtaining written consent from each surveyed student. Before completing the survey, the participants were informed of the study’s aim, their voluntary participation, anonymity, and ability to leave at any time. After excluding respondents who did not meet the inclusion criteria and incomplete surveys, 278 participants were enrolled in the study.

The obtained results were analysed using a structural index. Data were expressed as counts and percentages. The chi-square (χ^2^) test of independence was used to assess the relationships between the analysed variables. Statistical significance was set at *p* < 0.05. Statistical analyses were performed using STATISTICA (version 13.5).

## 3. Results

The sociodemographic characteristics of the respondents are presented in Table 1. In total, 278 respondents belonging to Generation Z were included in the study. The respondents were aged 18–28 years (mean age 20.7 ± 1.7; median 20). For further analysis, the participants were divided into two age groups: younger (aged 18–22 years) and older (aged 23–28 years). More than two-thirds (70.5%) of respondents were women. The participants came mainly from the Lublin Voivodeship (88.5%) and the Bialski District (75.2%) in southeastern Poland. Almost three-fifths (56.8%) did not travel abroad, and more than one-third (35.3%) travelled abroad once a year. Most participants reported average or low income (51.8% and 39.6%, respectively) (Table 1). Almost all respondents followed an omnivorous diet (84.3%) during the study, including those who were vegetarians in the past (5.9%) or were willing to limit meat in their diet (5.2%). Only four participants were pescovegetarians, followed by five vegetarians and one vegan. Regarding diet, for further analysis, the participants were divided into two groups: “meat-eaters”, including persons who eat meat and/or fish, and “meat-avoiders”, including vegans, vegetarians, and individuals who followed a vegetarian diet in the past or were able to limit meat in their diets. Meat avoiders were more often women than men (19.9% vs. 1.2%; *p* = 0.001) and health discipline students than non-medical students (19.9% vs. 9.2%; *p* = 0.011) (Table 2).

### 3.1. Attitudes Towards Cooking, New Dishes, and the Cuisines of Other Cultures

The results concerning attitudes towards cooking, new dishes, and cuisines of other cultures are presented in Table 3 and Table 4. Over half of the total respondents (54.3%) declared cooking for themselves every day, and more women than men did so (58.2% vs. 45.1%; *p* = 0.046) (Table 3). They occasionally (49.3%) or never (31.3%) watched TV culinary programs about world cuisines (Table 4). Less than one-fifth (19.1%) of the total participants were willing to try new dishes, with more respondents who often watched these programs than those who watched occasionally (37.0% vs. 19.7%; *p* = 0.0001) (Table 3 and Table 4). Nevertheless, more participants (63.0%) rarely tried new dishes, although they often watched these programs (Table 4). Three-quarters of the respondents occasionally visited ethnic restaurants. Men declared visiting them more regularly than women (35.4% vs. 19.9%, *p* = 0.006). No significant differences were found between visiting ethnic restaurants and other sociodemographic factors or trying new dishes.

### 3.2. Awareness of and Attitudes Towards Spices and Their Usage

The respondents pointed out family (28.9% of the total answers), the Internet (27.9%), and friends (16.7%) as sources of knowledge about the spices. They indicated taste (33.5%), flavour (22.6%), and curiosity (15.3%) as the main reasons for purchasing seasonings. A summary of the responses regarding awareness of and attitudes towards spices and their usage is presented in Table 3.

Most of the respondents (61.2%) declared using many spices (beyond salt and pepper), more often women than men (67.9% vs. 45.1%; *p* = 0.0004), and more often participants aged ≥23 years than the younger group aged ≤22 years (82.9% vs. 58.0%; *p* = 0.0048). Regarding diet, slightly more meat avoiders used many spices compared to meat eaters, but the differences were not statistically significant (67.5% vs. 60.1%; *p* = 0.373) (Table 3).

The majority of the total respondents stated that spices are necessary to add flavour to dishes (82.7%), both women and men, whereby female respondents agreed with this statement more often (86.2% vs. 72.0%; *p* = 0.0047) (Table 3).

The participants mentioned 55 seasonings used at least once a week (Supplementary Materials S1), the most common being pepper, including black, cayenne, black with powdered lemons, and herbal pepper mix (20.0% of the total answers). Although the question concerned spices, many of them pointed out salt in second place (16.8% of the total answers). The other most common seasonings were paprika (14.2%, including chili, sweet, and smoked), followed by basil (9.6%), oregano, and garlic (including fresh and granulated) (7.0% each), marjoram and spice mixes dedicated to various dishes (3.8% each), Provence herbs (3.2%), and curry (3.1%). The seasonings used less than once a week, but at least once a year, were paprika (including sweet and chili; 11.3% of the total answers), followed by curry (9.5%), spice mixes (7.3%), oregano (7.0%), cinnamon and turmeric (6.8% each), marjoram (5.2%), and basil (5.0%). The respondents listed 56 seasonings used, with the corresponding frequencies (Supplementary Materials S1).

Respondents who often watched culinary programs used more spices than those who watched them rarely or never (85.2%, 57.7%, and 51.7%, respectively; *p* = 0.0002) (Table 4). No significant differences were found between visiting ethnic restaurants and the frequency of spice use.

Respondents who had their own garden and grew spices (41.7%) used more spices than those who did not grow any of them (72.4% vs. 53.1%; *p* = 0.001) (Table 5). Most often, they pointed out basil (23.1% of the total answers), mint (14.5%), dill (11.4%), oregano (8.2%), parsley (10.6%), thyme (5.5%), lovage (3.9%), and garlic and rosemary (3.5% each).

Regarding medicinal properties, almost three-quarters (73.1%) of the participants recognised the health properties of spices in general or in selected spices. Participants aged ≥23 years perceived this effect more often than the younger group (82.8% vs. 71.6%), and more often for spices in general than participants aged ≤22 years (45.7% vs. 25.5%; *p* = 0.040). In turn, younger students attributed this feature more often to the selected ones (46.1%; *p* = 0.040). Regarding fields of study, health discipline students recognised the health properties of the majority of spices more often than non-medical students (33.1% vs. 23.2%). However, the differences were not statistically significant (*p* = 0.197) (Table 6).

Approximately one-fifth (21.6%) of the respondents declared using spices to improve their health, and nearly one-third (32.4%) used them preventively. Younger participants denied the use of spices for medicinal purposes more often than the older group, but the differences were not statistically significant (48.1% vs. 31.4%; *p* = 0.08). No statistically significant associations were found for other sociodemographic factors. In turn, participants with higher awareness of the health properties of spices used them more often to improve their health or preventively (42.3%, both; *p* < 0.000) (Table 6).

In addition, the respondents who grew their own spices declared the use of spices to improve their health more often than the rest of the participants (34.5% vs. 12.3%; *p* < 0.000). In turn, respondents who did not grow any spices declared use of them more often than participants who were gardening (35.2% vs. 28.4%; *p* < 0.000) (Table 5). The respondents identified 39 spices with health properties. The most frequently mentioned spices were ginger (19.0%), turmeric (16.3%), garlic (10.1%), cinnamon (6.6%), mint and cloves (5% each), marjoram (4.7%), pepper and black cumin (3.5% each), and oregano (3.1%).

## 4. Discussion

Herbs and spices are sources of bioactive compounds that provide health benefits beyond their basic nutritional values [6]. Due to their antioxidant, anti-inflammatory, and anti-carcinogenic properties, they may be an important addition to a balanced diet and help prevent non-communicable chronic diseases, such as obesity, cardiovascular diseases, diabetes, and cancers [6,14], which are serious public health problems worldwide [15]. However, public awareness of diet-related diseases and their risk factors remains insufficient [16]. In a study by Żarnowski et al. [16] conducted among Polish adults, the respondents most often recognised overweight/obesity (85.0%) out of eight diet-related diseases as disorders resulting from improper lifestyle, followed by diabetes mellitus (74.0%), arterial hypertension (68.2%), myocardial infarction (59.1%), and cancer (55.9%). Moreover, less than two-thirds (62.7%) of the participants correctly indicated too little consumption of vegetables and fruits, about half of them (53.7%) indicated excessive consumption of saturated fatty acids and trans isomers, and around two-fifths indicated too little consumption of fish, oils, and dietary fibre intake (43.6% and 38.6%, respectively) as diet-related risk factors. Participants with higher education and those suffering from chronic diseases were more aware of these issues [16]. Therefore, providing knowledge of diet-related diseases and dietary risk factors, as well as protective factors, such as bioactive food compounds, including herbs and spices [14,16], is necessary to improve the effectiveness of educational programs on eating behaviours. Prophylaxis of the aforementioned diseases is important, especially in early adulthood, which is often a period of moving out of a family home and leads to multiple life transitions, including environmental, social, and lifestyle changes, which may affect eating habits and persist later in life [17,18]. However, in our study, the place of origin of many respondents was the surrounding area, and the influence of familiar habits on their eating behaviour was observed.

Although the respondents declared that they used various spices, the most frequently mentioned seasonings were pepper, followed by salt. These seasonings were especially common in Poland during the Socialist period and in the second half of the 20th century because they were more available than other spices [7]. Therefore, their popularity might be similar to that of the present day. It is worth noting that spice mixes (e.g., for fish, meat, poultry, and potatoes), in which salt is often the main ingredient [19], were also popular among respondents. Although pepper spices have high antioxidant activity due to their piperine content, which may positively affect human health and longevity [20,21], high dietary sodium intake is a risk factor for the development of hypertension, cardiovascular diseases, gastric cancer, and nephrolithiasis, and leads to reduced bone mineral density and osteoporosis. Therefore, salt intake should be limited in the diet [22].

In turn, in the study by Gajewska et al. [23] conducted among Polish adults, the most frequently consumed herbs were basil, garlic, ginger, dill, marjoram, and parsley [23]. In a study by Łuczaj et al. [7] conducted among Polish respondents aged 18–85, who were more literate and interested in food and plants, the 10 most common seasonings were capsicum, black pepper, turmeric, oregano, cinnamon, marjoram, ginger, basil, thyme, and bay leaf. At the end of the 20th century, caraway was often added to white bread in Poland. Caraway is still a basic additive, next to salt, in the production of sauerkraut and mature pork sausages [24]. However, the consumption of bread and sauerkraut, which was previously a constant presence in traditional dinner dishes, has decreased significantly. The surveyed students did not have the habit on their own of using the spice of Slavic origin, which is caraway. In contrast to Romance-speaking populations, an interesting feature of Iranian, Turkish, Slavic, and Baltic Sea Region cuisine is the use of dill [25], which, however, did not find much response in the results of our study, apart from being listed as a plant grown in one’s own garden. Similar to the study by Łuczaj et al. [7], the use of saffron, nutmeg, and pimento in the kitchen is exceptional.

Although our respondents belonged to Generation Z, which has better access to information, travels more easily, and is more exposed to new concepts and various cultures, in our study, family, followed by the Internet and friends, were the main sources of knowledge about spices. Moreover, they were unwilling to try new dishes; three-quarters of them frequented ethnic restaurants occasionally, and almost three-fifths of them did not travel abroad. These findings may explain the low popularity of seasonings of world cuisines among our participants, because consumers’ readiness for new food experiences resulting from interest in global and ethnic food contributes to enhanced curiosity about flavour mixes and exotic spices [6]. Similarly, in the Kiciak et al. [26] study conducted among Polish female respondents, the most preferred world cuisine was Polish cuisine (77% of the respondents aged between 20 and 59 years old and 79% among older respondents), followed by Italian cuisine, which was more common among younger participants aged 20–30 and 31–59 (79% and 72%, respectively), compared to 28% of the participants aged 60–79 [26]. In Poland, students from rural areas, especially those located on the outskirts of the country, as is the case with the majority of the participants in our study, are more prone to developing depression and undertake fewer tourist activities, both domestic and foreign, and the closed environment of their origin prevents them from opening up to learning about other cultures through couch surfing [27,28,29].

More than half of the respondents declared that they cooked for themselves every day. This is important because culinary practices determine dietary quality and overall health outcomes. Proper home-cooked meal preparation skills, including spice usage, may be associated with better dietary quality. For instance, young Spanish adults with healthy culinary habits, such as home cooking, and healthy culinary knowledge, including confidence in culinary skills, demonstrated healthier eating behaviours [30]. In addition, educational interventions, including the development of practical food knowledge and an increase in culinary skills through a combined lecture and hands-on cooking class format, might improve cooking self-efficacy and behaviours related to cooking and healthy eating [31]. In contrast, Isbill et al. [32] found that participants who ate out of the home more frequently were less likely to use spices daily. Fritts et al. [33] showed that rural students from Pennsylvania State University consume plain over seasoned vegetables because of their aversion to novel herbs and spices. However, repeated exposure to seasonings in school lunch programs devoted to youths with family incomes at 130% or below the poverty level (free lunch) or with incomes between 130–185% above the poverty level (reduced-price lunch) has the potential to boost the acceptance of offerings with herbs and spices. Both economic and cultural support may be necessary to change eating habits, as research by Gutschall et al. [34] has shown among the conservative rural American Rust Belt population.

In general, taste and flavour were the main attributes of seasonings for participants. The most recognisable benefits of herbs and spices might contribute to the greater consumption of herbs and spices. In addition, enhancing the multisensory flavour experience may lead to a reduction in sodium intake [6].

Our respondents did not systematically use spices that they considered to have healing effects (except garlic and marjoram). Similarly, in a study conducted among Australian adults, sensory preferences were important motivators for using herbs and spices [35]. Regarding healing properties, in our research, older groups of respondents paid attention to these issues. In addition, respondents who grew their own spices more often declared that they used them to improve their health. It is worth noting that the health effects of compounds included in herbs and spices may be imperceptible over relatively short periods, including months or a few years, whereas their consumption throughout life as part of the daily healthy diet might have a significantly beneficial effect on the human body [6]; therefore, educational curricula should also increase awareness of this issue, especially among younger participants. In addition, demonstration herb gardens on university campuses are a valuable initiative [36].

It is worth noting that pro-healthy plant-based diets, including the Mediterranean diet, widely recommend the use of herbs and spices [1,37]. Mediterranean ingredients such as extra virgin olive oil, fresh garlic, and potted basil appear to be the most prominent taste markers in salads and dinner patterns of the Western middle class [38]. In our study group, basil was more often indicated as the preferred spice than garlic was. It is not surprising that the most commonly cultivated spice plant in one’s own garden was basil.

Most of the respondents followed an omnivorous diet. Only six participants followed a vegan or vegetarian diet. This may be due to socio-demographic conditions. Borowiec and Aranowska [39] defined three nutritional styles of Poles: “careless”, “healthy”, and “traditional”, and their socio-demographic determinants. Men, younger people, and those living in larger households followed a “careless” style. In turn, the “healthy” style was characteristic of women, those with tertiary and secondary education, students, and entrepreneurs. Younger participants living in smaller households, with secondary and vocational education, as well as residents of cities with a population between 50 and 200 thousand, represented the “traditional” style [39]. In addition, residents of large cities in Poland and emigrants in London, United Kingdom, avoid meat more often (6.7% and 16.1%, respectively) [40]. Our respondents constituted a homogeneous conservative community living in a small town, where sociocultural aspects learned from the family home may have a greater impact on their diet. In addition, most of them declared an average or low income, whereas the cost of vegetarian and vegan diets is often perceived as a barrier to implementing these recommendations [41]. In contrast, in a study conducted among Generation Z from Greece, India, and the United Kingdom, most of the respondents were willing to adopt a plant-based diet [42], which might be connected to the greater use of seasoning [23,37]. Gajewska et al. [23] observed a greater intake of seasonings among vegans and vegetarians than omnivores [23]. In turn, Wilson et al. [35] linked the regular usage of herbs and spices to higher adherence to the Mediterranean diet [35].

The limitation of the present study is the population surveyed. The study was conducted at a single university in southeast Poland, in a small town, where beliefs and traditions might also affect eating behaviours. Therefore, attributing such results to all Polish students or young Polish adults should be done cautiously.

## 5. Conclusions

Spices, because of their bioactive compounds, may constitute a valuable part of a balanced diet with beneficial health effects. Though they are most often used for their taste and flavour, it is worth taking advantage of their simultaneous health-promoting properties. Education about the composition and use of seasoning, as well as cooking skill improvement programs, connecting a combined lecture and hands-on cooking class format [31], is important, especially for younger generations, who are moving away from their family homes and starting to prepare their own meals. Currently, exposure to world cuisine is notable. Nevertheless, in small, traditional communities, attachment to familiar recipes may be observed. Therefore, promoting traditional cuisines and their characteristic seasonings, such as traditional Polish cuisine, before the transformation accompanying the entry of Poland into the orbit of the Soviet Union, a centrally managed economy with permanent shortages of non-essential products and a lack of foreign currency for food imports, which includes various herbs and spices, might help increase their intake [7,8]. Culinary educational programs aimed at increasing knowledge of the properties and usage of herbs and spices should also enhance interest in world cuisines, in addition to native cuisine, because it might lead to the inclusion of other, previously unknown spices [6]. In turn, a properly planned strategy for selecting ingredients for preparing meals in university canteens can help change culinary preferences [33]. In addition, shifting to plant-based diets may result in greater use of spices [23,35].

## Figures and Tables

**Table 1:** Sociodemographic characteristics of the respondents.

Characteristic		Frequency (*n*)	Percentage (%)
Total respondents (N)		278	100
Gender	female	196	70.5
	male	82	29.5
Age	18–22	243	87.4
	23–28	35	12.6
Field of education	Economic Sciences	142	51.1
	Health Sciences	136	48.9
Place of origin	Lublin Voivodeship	246	88.5
	Mazovian Voivodeship	21	7.6
	Podlaskie Voivodeship	10	3.6
	Pomeranian Voivodeship	1	0.4
Income	high	20	7.2
	average	144	51.8
	low	110	39.6
	no data	4	1.4

**Table 2:** Dietary patterns of the respondents.

	N	Meat-Eaters		Meat-Avoiders		*p*-Value *	Chi-Squared (χ^2^)
		*n*	%	*n*	%		
Total respondents	278	238	85.6%	40	14.4%		
**Gender**							
female	196	157	80.1%	39	19.9%	0.001	14.89
male	82	81	98.8%	1	1.2%		
**Age**							
18–22	243	208	85.6%	35	14.4%	0.985	0.0003
23–28	35	30	85.7%	5	14.3%		
**Fields of education**							
Economic Sciences	142	129	90.8%	13	9.2%	0.011	6.454
Health Sciences	136	109	80.1%	27	19.9%		

* Statistically significant *p* ≤ 0.05.

**Table 3:** Respondents’ attitudes towards cooking and spices.

		How Often Do You Cook For Yourself?				*p*-Value *	Chi-Squared (χ^2^)	How Often Do You Try New Dishes?				*p*-Value *	Chi-Squared (χ^2^)
		Every Day		Every Now and Then/ I Don’t Cook				Often		Rarely or Never			
	N	*n*	%	*n*	%			*n*	%	*n*	%		
Total Respondents	278	151	54.3%	127	45.7%			53	19.1%	225	80.9%		
Gender													
women	196	114	58.2%	82	41.8%	**0.0465**	3.963	40	20.4%	156	79.6%	0.378	0.777
men	82	37	45.1%	45	54.9%			13	15.9%	69	84.1%		
Age													
18–22	243	129	53.1%	114	46.9%	0.278	1.177	43	17.7%	200	82.3%	0.126	2.345
23–28	35	22	62.9%	13	37.1%			10	28.6%	25	71.4%		
Dietary pattern													
meat-eaters	238	134	56.3%	104	43.7%	0.105	2.629	46	19.3%	192	80.7%	0.785	0.0741
meat-avoiders	40	17	42.5%	23	57.5%			7	17.5%	33	82.5%		
		**Do you use spices beyond** **salt and pepper?**				***p*** -Value *	**Chi-Squared (χ^2^)**	**Do you think spices** **are essential for adding** **flavour to dishes?**				***p*** -Value *	**Chi-Squared (χ^2^)**
		**Yes, a lot**		**A few/never**				**Yes**		**It depends** **on the dish/No**			
	**N**	***n***	**%**	***n***	**%**			***n***	**%**	***n***	**%**		
**Total** **respondents**	**278**	**170**	**61.2%**	**108**	**38.8%**			**228**	**82.0%**	**50**	**18.0%**		
Gender													
women	196	133	67.9%	63	32.1%	**0.0004**	12.579	169	86.2%	27	13.8%	**0.005**	7.985
men	82	37	45.1%	45	54.9%			59	72.0%	23	28.0%		
Age													
18–22	243	141	58.0%	102	42.0%	**0.005**	7.941	199	81.9%	44	18.1%	0.8896	0.019
23–28	35	29	82.9%	6	17.1%			29	82.9%	6	17.1%		
Dietary pattern													
meat-eaters	238	143	60.1%	95	39.9%	0.373	0.793	195	81.9%	43	18.1%	0.931	0.007
meat-avoiders	40	27	67.5%	13	32.5%			33	82.5%	7	17.5%		

* Statistically significant *p* ≤ 0.05.

**Table 4:** Watching TV culinary programs vs. trying new dishes and using spices.

	How Often Do You Watch Culinary TV Programmes About the Cuisines of Other Cultures?						*p*-Value *	Chi-Squared (χ^2^)
	Often		Occasionally		I Don’t Watch			
N = 278	*n*	%	*n*	%	*n*	%		
	54	19.4%	137	49.3%	87	31.3%		
**How often do you try new dishes?**								
Often	20	37.0%	27	19.7%	6	6.9%	**0.0001**	19.689
Rarely/never	34	63.0%	110	80.3%	81	93.1%		
**Do you use any spices other than salt and pepper?**								
Yes, a lot	46	85.2%	79	57.7%	45	51.7%	**0.0002**	17.086
A few/never	8	14.8%	58	42.3%	42	48.3%		

* Statistically significant *p* ≤ 0.05.

**Table 5:** Gardening vs. usage of spices by the respondents.

	Do You Grow Spices in Your Garden?					
	Yes		No		*p*-Value *	Chi-Squared (χ^2^)
N = 278	*n*	%	*n*	%		
	116	41.7%	162	58.3%		
**Do you use any spices other than salt and pepper?**						
Yes, a lot	84	72.4%	86	53.1%	**0.001**	10.629
A few/sometimes or never	32	27.6%	76	46.9%		
**Do you use spices in your diet to improve your health?**						
Yes	40	34.5%	20	12.3%	**<0.00**	19.778
Preventively	33	28.4%	57	35.2%		
No	43	37.1%	85	52.5%		

* Statistically significant *p* ≤ 0.05.

**Table 6:** Awareness of and attitudes towards the health properties of spices among respondents.

		Do You Think That Spices Have Medicinal Properties?						*p*-Value *	Chi-Squared (χ^2^)	Do You Use Spices in Your Diet to Improve Your Health?						*p*-Value *	Chi-Squared (χ^2^)
		Yes		Some of Them		I Don’t Know/No				Yes		Preventively		No			
Total Respondents	N	*n*	%	*n*	%	*n*	%			*n*	%	*n*	%	*n*	%		
	278	78	28.1%	125	45.0%	75	27.0%			60	21.58%	90	32.37%	128	46.04%		
Gender																	
women	196	57	29.1%	89	45.4%	50	25.5%	0.667	0.808	43	21.9%	61	31.1%	92	46.9%	0.79	0.48
men	82	21	25.6%	36	43.9%	25	30.5%			17	20.7%	29	35.4%	36	43.9%		
Age																	
18–22	243	62	25.5%	112	46.1%	69	28.4%	**0.0402**	6.4297	53	21.8%	73	30.0%	117	48.1%	0.0762	5.15
23–28	35	16	45.7%	13	37.1%	6	17.1%			7	20.0%	17	48.6%	11	31.4%		
Fields of education																	
health sciences	142	33	23.2%	67	47.2%	42	29.6%	0.1971	3.248	27	19.0%	51	35.9%	64	45.1%	0.35	2.07
economic sciences	136	45	33.1%	58	42.6%	33	24.3%			33	24.3%	39	28.7%	64	47.1%		
Dietary pattern																	
meat-eaters	238	68	28.6%	105	44.1%	65	27.3%	0.178	3.446	54	22.7%	79	33.2%	105	44.1%	0.27	2.61
meat-avoiders	40	10	25.0%	20	50.0%	10	25.0%			6	15.0%	11	27.5%	23	57.5%		
**Do you use spices in your diet to improve your health?**																	
Yes	60	33	42.3%	23	18.4%	4	5.3%	**<0.00**	65.087								
Preventively	90	33	42.3%	44	35.2%	13	17.3%										
No	128	12	15.4%	58	46.4%	58	77.3%										

* Statistically significant *p* ≤ 0.05.

## Data Availability

The original contributions presented in this study are included in the article/Supplementary Materials. Further inquiries can be directed to the corresponding author(s).

## Supplementary Materials

The following supporting information can be downloaded at: https://www.mdpi.com/article/10.3390/nu18132139/s1, Supplementary Materials S1. Frequencies of consumption of specific seasonings.
