# Supplementary material for: Uses of Spices Amongst Generation Z Students at a University Located in Rural Poland: An Exploratory Study
Source: Nutrients. 2026 Jul 2;18(13):2139. doi: 10.3390/nu18132139 (PMC13363361; doi:10.3390/nu18132139)
Supplement: Supplementary file 1 [file nutrients-18-02139-s001.zip › nutrients-4372123-supplementary.pdf]

Supplementary Materials S1. *Frequencies of consumption of specific seasonings.*

Type of seasoning	Seasonings used at least once a week (N=55)		Seasonings used less than once a week, but at least once a year (N=56)	
	Reporting intake			
	n	%	n	%
**Adjika**	-	-	1	0.2%
**Allspice**	2	0.2%	8	1.8%
**Anise**	-	-	1	0.2%
**Basil**	87	9.6%	22	5.0%
**Bay leef**	2	0.2%	7	1.6%
**Black cumin**	1	0.1%	1	0.2%
**Caraway seed**	3	0.3%	9	2.0%
**Cardamom**	2	0.2%	3	0.7%
**Chamomile**	-	-	1	0.2%
**Cinnamon**	18	2.0%	30	6.8%
**Cloves**	5	0.5%	14	3.2%
**Coriander**	1	0.1%	5	1.1%
**Cumin**	1	0.1%	9	2.0%
**Curry mixes**	28	3.1%	42	9.5%
**Dill**	4	0.4%	5	1.1%
**Dried tomatos**	3	0.3%	1	0.2%
**Fenugreek**	-	-	1	0.2%
**Garlic, including granulated garlic**	64	7.0%	9	2.0%
**Garlic with spices**	1	0.1%	-	-
**Ginger**	10	1.1%	13	2.9%
**Gochujang**	-	-	1	0.2%
**Green savoy**	1	0.1%	-	-
**Juniper**	-	-	2	0.5%
**Lovage**	1	0.1%	1	0.2%
**Marjoram**	35	3.8%	23	5.2%
**Mint**	5	0.5%	2	0.5%
**Mustard**	-	-	2	0.5%
**Nutmeg**	1	0.1%	7	1.6%
**Oregano**	64	7.0%	31	7.0%
**Paprika, including:**	129	14.2%	50	11.3%
Chilli	42	4.6%	26	5.9%
Sweet paprika	76	8.4%	24	5.4%
Smoked paprika	11	1.2%	-	-
**Parsley**	3	0.3%	1	0.2%
**Pepper, including:**	182	20.0%	17	3.9%
Black pepper	157	17.3%	6	1.4%
Cayenne pepper	5	0.5%	6	1.4%
Black pepper with lemon powder	1	0.1%	3	0.7%
Pepper mixes	1	0.1%	-	-
Pieprz ziołowy ("Herbal pepper mix")	18	2.0%	2	0.5%

**Provance herb mix**	29	3.2%	15	3.4%
**Rosemary**	2	0.2%	15	3.4%
**Saffron**	1	0.1%	-	-
**Sage**	-	-	1	0.2%
**Salt**	153	16.8%	7	1.6%
**Salt with garlic**	1	0.1%	-	-
**Seasalt**	-	-	1	0.2%
**Savory**	1	0.1%	-	-
**Spice mixes, including:**	34	3.8%	32	7.3%
Gyros mix	11	1.2%	3	0.7%
Przyprawa korzenna ("Gingerbread spice)	-	-	3	0.7%
Spices for cakes	-	-	4	0.9%
Spice mix for chicken	4	0.4%	2	0.5%
Spice mix for eggs	1	0.1%	-	-
Spice mix for fish	1	0.1%	2	0.5%
Spice mix for kebab	-	-	1	0.2%
Spice mix for meat	8	0.9%	2	0.5%
Spice mix for pizza	1	0.1%	2	0.5%
Spice mix for salads	1	0.1%	-	-
Spice mix for potatoes	6	0.7%	9	2.0%
Spice mix for soups	1	0.1%	3	0.7%
**Soup seasoning, including:**	12	1.3%	8	1.8%
Jarzynka ("Dried vegetables")	-	-	1	0.2%
Vegeta ™ brand	7	0.7%	2	0.5%
Maggi spice	-	-	2	0.5%
Kucharek ™ brand	3	0.3%	1	0.2%
Ziarenka smaku ("Grains of flavor")	2	0.2%	1	0.2%
Stock cubes	-	-	1	0.2%
**Sugar**	2	0.2%	-	-
**Tarragon**	2	0.2%	-	-
**Thyme**	6	0.7%	14	3.2%
**Turmeric**	12	1.3%	30	6.8%
**Vanilla**	1	0.1%	-	-
**Wild garlic**	1	0.1%	-	-
**Total:**	910		441	
